# 4-Ammonio-2,2,6,6-tetra­methyl­piperidinium bis­(dihydrogen phosphate) monohydrate

**DOI:** 10.1107/S1600536809008563

**Published:** 2009-03-14

**Authors:** Mohamed Lahbib Mrad, Sameh Akriche, Mohamed Rzaigui, Cherif Ben Nasr

**Affiliations:** a Laboratoire de Chimie des Matériaux, Faculté des Sciences de Bizerte, 7021 Zarzouna, Tunisia

## Abstract

In the crystal structure of the title compound, C_9_H_22_N_2_^2+^·2H_2_PO_4_^−^·H_2_O, the H_2_PO_4_^−^ anions are hydrogen bonded to each other, forming a ribbon parallel to the *b* axis. The water mol­ecules connect these ribbons *via* O—H⋯O hydrogen bonds. The organic cations are attached to the dihydrogen phosphate anions and water mol­ecules through N—H⋯O and C—H⋯O hydrogen bonds, forming an infinite three-dimensional network.

## Related literature

For common applications of hybrid compounds, see: Wang *et al.* (1996 ▶); Coombs *et al.* (1997 ▶); Masse *et al.* (1993 ▶). For organic phosphates, see: Baoub & Jouini (1998 ▶). For a discussion of the O⋯O distances, see: Kefi *et al.* (2006 ▶). For P⋯O bond-length data, see: Oueslati & Ben Nasr (2006 ▶). For the [(H_2_PO_4_^−^)_4_]_*n*_ subnetwork as a polyanion, see: Kefi *et al.* (2006 ▶).
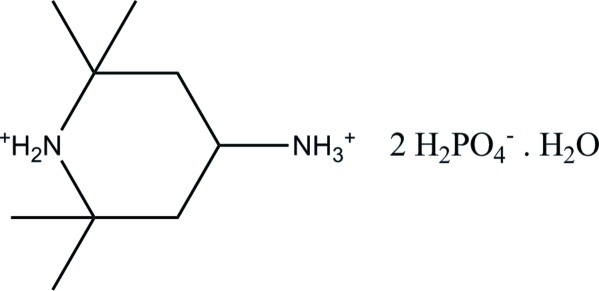


## Experimental

### 

#### Crystal data

C_9_H_22_N_2_^2+^·2H_2_PO_4_^−^·H_2_O
*M* *_r_* = 370.27
Monoclinic, 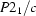

*a* = 12.604 (5) Å
*b* = 8.249 (2) Å
*c* = 16.321 (2) Å
β = 104.56 (4)°
*V* = 1642.4 (8) Å^3^
*Z* = 4
Mo *K*α radiation
μ = 0.31 mm^−1^
*T* = 298 K
0.5 × 0.35 × 0.25 mm

#### Data collection

Enraf–Nonius TurboCAD-4 diffractometer
Absorption correction: none
6617 measured reflections
3953 independent reflections
2575 reflections with *I* > 2σ(*I*)
*R*_int_ = 0.056
2 standard reflections frequency: 120 min intensity decay: 8%

#### Refinement

*R*[*F* ^2^ > 2σ(*F* ^2^)] = 0.049
*wR*(*F* ^2^) = 0.128
*S* = 1.00
3953 reflections
216 parameters
3 restraints
H atoms treated by a mixture of independent and constrained refinement
Δρ_max_ = 0.32 e Å^−3^
Δρ_min_ = −0.49 e Å^−3^

### 

Data collection: *CAD-4 EXPRESS* (Enraf–Nonius, 1994 ▶); cell refinement: *CAD-4 EXPRESS*; data reduction: *XCAD4* (Harms & Wocadlo, 1995 ▶); program(s) used to solve structure: *SHELXS97* (Sheldrick, 2008 ▶); program(s) used to refine structure: *SHELXL97* (Sheldrick, 2008 ▶); molecular graphics: *ORTEP-3 for Windows* (Farrugia, 1997 ▶); software used to prepare material for publication: *WinGX* (Farrugia, 1999 ▶).

## Supplementary Material

Crystal structure: contains datablocks I, global. DOI: 10.1107/S1600536809008563/wk2099sup1.cif

Structure factors: contains datablocks I. DOI: 10.1107/S1600536809008563/wk2099Isup2.hkl

Additional supplementary materials:  crystallographic information; 3D view; checkCIF report

## Figures and Tables

**Table 1:** Hydrogen-bond geometry (Å, °)

*D*—H⋯*A*	*D*—H	H⋯*A*	*D*⋯*A*	*D*—H⋯*A*
O2—H2⋯O1^i^	0.82	1.74	2.537 (3)	163
O4—H4⋯O5^ii^	0.82	1.79	2.538 (3)	152
O6—H6⋯O3	0.82	1.83	2.646 (3)	172
O7—H7⋯O1	0.82	1.85	2.662 (3)	173
O9—H91⋯O5	0.85 (1)	1.97 (1)	2.811 (3)	170 (3)
O9—H92⋯O3^iii^	0.85 (1)	2.00 (1)	2.837 (3)	165 (5)
O9—H92⋯O4^iii^	0.85 (1)	2.66 (5)	3.256 (3)	128 (5)
N1—H1*A*⋯O8^iv^	0.90	1.84	2.742 (3)	176
N1—H1*B*⋯O5^v^	0.90	2.31	3.168 (3)	159
N1—H1*B*⋯O8^v^	0.90	2.33	3.038 (3)	136
N2—H2*A*⋯O2	0.89	2.05	2.929 (3)	172
N2—H2*A*⋯O1	0.89	2.51	3.076 (3)	122
N2—H2*B*⋯O3^ii^	0.89	2.07	2.919 (3)	160
N2—H2*C*⋯O9	0.89	1.88	2.721 (4)	156
C3—H3⋯O6^ii^	0.98	2.59	3.406 (3)	141
C4—H4*B*⋯O9^vi^	0.97	2.58	3.492 (4)	158
C9—H9*A*⋯O7^vi^	0.96	2.40	3.343 (3)	168

Symmetry codes: (i) 
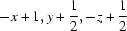
; (ii) 
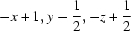
; (iii) 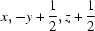; (iv) 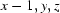; (v) 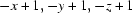; (vi) 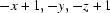.

**Fig. 1.**
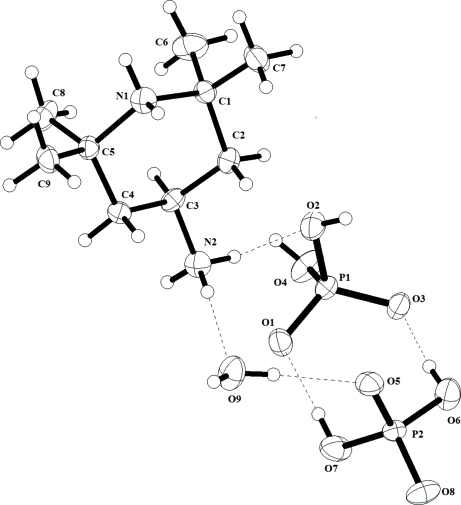
A view of (C9H28N2O9P2), showing 40% probability displacement ellipsoids and arbitrary spheres for the H atoms.

**Fig. 2.**
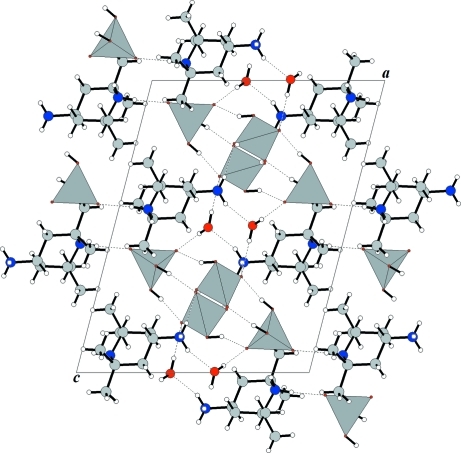
Projection of (C9H28N2O9P2) subnetwork along the b axis.

## supplementary crystallographic information

### Comment

The combination of organic molecules and inorganic materials was the starting point for the development of new hybrid compounds having many practical and potential applications in various fields, such as biomolecular sciences, catalysis, fuel cells, liquid crystal-material development and quadratic nonlinear optics (Wang *et al.*, 1996; Coombs *et al.*, 1997; Masse *et al.*, 1993). Among these hybrid compounds, organic phosphates are particularly interesting owing to the specific H-bond schemes that they can present in their infinite networks (Baoub & Jouini, 1998). We report here the synthesis and the crystal structure of a new member of this family, the compound (C_9_H_28_N_2_O_9_P_2_). As shown in Fig. 1, to ensure charge equilibrium the organic species is doubly protonated at N1 and N2 nitrogen atoms. Thus, the structure associates to each 4-ammonio-2,2,6,6,-tetramethylpiperidinium cation two dihydrogen phosphate anions and one water molecule. The two H_2_PO_4_^-^ are crystallographically independent. They form, *via* H-bonds a repetitive motif of four member (H_2_PO_4_^-^)_4_ (Fig. 2). The organic cations and the water molecules are attached to these units *via* (O—H···O), N—H···O and C—H···O hydrogen bonds to perform a three dimensional infinite network. An examination of the anionic entity shows that the O···O distances involved in hydrogen bonds [2.537 (3) to 2.662 (3) A°] are close to the O···O distances in the H_2_PO_4_^-^ tetrahedra [2.469 (3) to 2.536 (3) A°], so one could consider the [(H_2_PO_4_^-^)_4_]_n_ subnetwork as a polyanion (Kefi *et al.*, 2OO6). The detailed geometries of H_2_P(1)O_4_^-^ and H_2_P(2)O_4_^-^ entities show that the P···O distances significantly are shorter [1.480 (2) to 1.515 (2) A°] than the P···OH distances [1.552 (2) to 1.582 (2) A°], which is in full agreement with those observed in such anions in other organic dihydrogenomonophosphates [Oueslati and Ben Nasr, 2006].

### Experimental

Crystals of the title compound have been prepared in a Petri dish by adding 50 mmol of concentrated orthophosphoric acid (Fluka, 85%, d = 1.7) to 25 mmol of 4-Amino-2,2,6,6-tetramethylpiperidine (Acros) dissolved in ethanol. After agitation, the resulting solution has been slowly evaporated at room temperature until the formation of single crystals suitable for X-ray structure analysis and these remained stable under normal conditions of temperature and humidity.

### Refinement

Hydrogen atoms were placed in calculated positions and refined as part of a riding model except those of the water molecule which were located in difference Fourier maps and their positions and isotropic displacement parameters refined.

### Crystal data

**Table:**

C_9_H_22_N_2_^2+^·2H_2_PO_4_^−^·H_2_O	*F*(000) = 792
*M**_r_* = 370.27	*D*_x_ = 1.497 Mg m^−^^3^
Monoclinic, *P*2_1_/*c*	Mo *K*α radiation, λ = 0.71073 Å
Hall symbol: -P 2ybc	Cell parameters from 25 reflections
*a* = 12.604 (5) Å	θ = 9–11°
*b* = 8.249 (2) Å	µ = 0.31 mm^−^^1^
*c* = 16.321 (2) Å	*T* = 298 K
β = 104.56 (4)°	Prism, colorless
*V* = 1642.4 (8) Å^3^	0.5 × 0.35 × 0.25 mm
*Z* = 4	

### Data collection

**Table:**

Enraf–Nonius TurboCAD-4 diffractometer	*R*_int_ = 0.056
Radiation source: fine-focus sealed tube	θ_max_ = 28.0°, θ_min_ = 2.6°
graphite	*h* = −16→16
Nonprofiled ω scans	*k* = 0→10
6617 measured reflections	*l* = −10→21
3953 independent reflections	2 standard reflections every 120 min
2575 reflections with *I* > 2σ(*I*)	intensity decay: 8%

### Refinement

**Table:**

Refinement on *F*^2^	Primary atom site location: structure-invariant direct methods
Least-squares matrix: full	Secondary atom site location: difference Fourier map
*R*[*F*^2^ > 2σ(*F*^2^)] = 0.049	Hydrogen site location: inferred from neighbouring sites
*wR*(*F*^2^) = 0.128	H atoms treated by a mixture of independent and constrained refinement
*S* = 1.00	*w* = 1/[σ^2^(*F*_o_^2^) + (0.0646*P*)^2^] where *P* = (*F*_o_^2^ + 2*F*_c_^2^)/3
3953 reflections	(Δ/σ)_max_ = 0.001
216 parameters	Δρ_max_ = 0.32 e Å^−^^3^
3 restraints	Δρ_min_ = −0.48 e Å^−^^3^

### Special details

**Table:**

Geometry. All e.s.d.'s (except the e.s.d. in the dihedral angle between two l.s. planes) are estimated using the full covariance matrix. The cell e.s.d.'s are taken into account individually in the estimation of e.s.d.'s in distances, angles and torsion angles; correlations between e.s.d.'s in cell parameters are only used when they are defined by crystal symmetry. An approximate (isotropic) treatment of cell e.s.d.'s is used for estimating e.s.d.'s involving l.s. planes.
Refinement. Refinement of *F*^2^ against ALL reflections. The weighted *R*-factor *wR* and goodness of fit *S* are based on *F*^2^, conventional *R*-factors *R* are based on *F*, with *F* set to zero for negative *F*^2^. The threshold expression of *F*^2^ > σ(*F*^2^) is used only for calculating *R*-factors(gt) *etc*. and is not relevant to the choice of reflections for refinement. *R*-factors based on *F*^2^ are statistically about twice as large as those based on *F*, and *R*- factors based on ALL data will be even larger.

### Fractional atomic coordinates and isotropic or equivalent isotropic displacement parameters (Å^2^)

**Table:**

	*x*	*y*	*z*	*U*_iso_*/*U*_eq_	
P1	0.52437 (5)	0.19580 (8)	0.19702 (4)	0.02151 (17)	
P2	0.79116 (5)	0.36565 (8)	0.37537 (4)	0.02164 (17)	
O1	0.57523 (14)	0.0956 (2)	0.27473 (12)	0.0301 (4)	
O2	0.42447 (15)	0.2885 (2)	0.21633 (14)	0.0332 (5)	
H2	0.4379	0.3859	0.2203	0.050*	
O3	0.60240 (14)	0.3112 (2)	0.17133 (12)	0.0279 (4)	
O4	0.47608 (15)	0.0832 (3)	0.12059 (12)	0.0372 (5)	
H4	0.4155	0.0509	0.1233	0.056*	
O5	0.70615 (14)	0.4348 (2)	0.41668 (12)	0.0312 (5)	
O6	0.77241 (16)	0.4361 (3)	0.28269 (12)	0.0356 (5)	
H6	0.7172	0.3954	0.2520	0.053*	
O7	0.77502 (16)	0.1773 (2)	0.36442 (15)	0.0380 (5)	
H7	0.7113	0.1577	0.3394	0.057*	
O8	0.90532 (14)	0.3991 (3)	0.42314 (13)	0.0358 (5)	
O9	0.59416 (18)	0.2150 (3)	0.49650 (14)	0.0393 (5)	
H91	0.626 (3)	0.290 (3)	0.476 (2)	0.055 (11)*	
H92	0.597 (4)	0.226 (6)	0.5489 (11)	0.13 (2)*	
N1	0.12655 (16)	0.3672 (3)	0.44120 (13)	0.0195 (4)	
H1A	0.0535	0.3763	0.4329	0.023*	
H1B	0.1573	0.4360	0.4833	0.023*	
N2	0.40484 (17)	0.1521 (3)	0.37758 (15)	0.0301 (5)	
H2A	0.4183	0.1931	0.3307	0.045*	
H2B	0.4093	0.0445	0.3764	0.045*	
H2C	0.4540	0.1900	0.4226	0.045*	
C1	0.15485 (19)	0.4285 (3)	0.36124 (16)	0.0228 (5)	
C2	0.2733 (2)	0.3797 (3)	0.36452 (18)	0.0268 (6)	
H22A	0.2892	0.4053	0.3108	0.032*	
H22B	0.3231	0.4418	0.4083	0.032*	
C3	0.29204 (19)	0.2008 (3)	0.38261 (17)	0.0241 (5)	
H3	0.2383	0.1395	0.3400	0.029*	
C4	0.27523 (19)	0.1613 (3)	0.46967 (17)	0.0233 (5)	
H4A	0.3253	0.2256	0.5121	0.028*	
H4B	0.2919	0.0478	0.4823	0.028*	
C5	0.15786 (19)	0.1958 (3)	0.47367 (17)	0.0221 (5)	
C6	0.0747 (2)	0.3618 (4)	0.28233 (18)	0.0404 (8)	
H6A	0.0866	0.2475	0.2781	0.061*	
H6B	0.0862	0.4155	0.2331	0.061*	
H6C	0.0010	0.3804	0.2862	0.061*	
C7	0.1434 (2)	0.6124 (3)	0.36298 (19)	0.0345 (7)	
H7A	0.0700	0.6401	0.3646	0.052*	
H7B	0.1589	0.6580	0.3131	0.052*	
H7C	0.1941	0.6550	0.4123	0.052*	
C8	0.0775 (2)	0.0728 (4)	0.4223 (2)	0.0339 (7)	
H8A	0.0875	0.0665	0.3660	0.051*	
H8B	0.0038	0.1062	0.4198	0.051*	
H8C	0.0908	−0.0317	0.4488	0.051*	
C9	0.1488 (2)	0.1962 (3)	0.56518 (17)	0.0291 (6)	
H9A	0.1607	0.0885	0.5879	0.044*	
H9B	0.0771	0.2325	0.5670	0.044*	
H9C	0.2030	0.2679	0.5982	0.044*	

### Atomic displacement parameters (Å^2^)

**Table:**

	*U*^11^	*U*^22^	*U*^33^	*U*^12^	*U*^13^	*U*^23^
P1	0.0200 (3)	0.0239 (3)	0.0216 (3)	−0.0024 (3)	0.0070 (3)	−0.0030 (3)
P2	0.0183 (3)	0.0229 (3)	0.0226 (4)	0.0007 (3)	0.0032 (2)	−0.0014 (3)
O1	0.0333 (10)	0.0257 (10)	0.0286 (11)	−0.0049 (8)	0.0030 (8)	0.0036 (8)
O2	0.0323 (10)	0.0254 (10)	0.0473 (13)	0.0010 (8)	0.0203 (9)	−0.0007 (10)
O3	0.0245 (9)	0.0328 (10)	0.0272 (10)	−0.0066 (8)	0.0077 (8)	0.0008 (9)
O4	0.0286 (10)	0.0517 (13)	0.0333 (12)	−0.0120 (9)	0.0115 (9)	−0.0202 (10)
O5	0.0281 (9)	0.0362 (11)	0.0317 (11)	0.0093 (8)	0.0121 (8)	0.0049 (9)
O6	0.0392 (11)	0.0427 (12)	0.0228 (11)	−0.0127 (9)	0.0037 (8)	0.0015 (9)
O7	0.0322 (10)	0.0249 (10)	0.0506 (14)	0.0027 (8)	−0.0014 (10)	−0.0035 (10)
O8	0.0209 (9)	0.0438 (12)	0.0382 (12)	−0.0010 (8)	−0.0009 (8)	−0.0108 (10)
O9	0.0422 (12)	0.0495 (14)	0.0278 (12)	−0.0122 (10)	0.0117 (10)	−0.0011 (11)
N1	0.0193 (9)	0.0214 (10)	0.0183 (11)	0.0005 (8)	0.0059 (8)	0.0003 (9)
N2	0.0263 (11)	0.0339 (13)	0.0335 (13)	0.0045 (9)	0.0136 (10)	−0.0046 (11)
C1	0.0231 (12)	0.0300 (13)	0.0154 (12)	0.0004 (11)	0.0051 (10)	0.0048 (11)
C2	0.0252 (12)	0.0308 (14)	0.0275 (15)	−0.0008 (11)	0.0122 (11)	0.0038 (12)
C3	0.0194 (11)	0.0283 (13)	0.0268 (14)	−0.0006 (10)	0.0100 (10)	−0.0038 (12)
C4	0.0227 (11)	0.0209 (13)	0.0263 (14)	0.0032 (10)	0.0064 (10)	0.0010 (11)
C5	0.0226 (11)	0.0194 (11)	0.0255 (14)	−0.0010 (10)	0.0082 (10)	−0.0001 (11)
C6	0.0330 (15)	0.061 (2)	0.0229 (15)	0.0037 (14)	−0.0001 (12)	−0.0019 (15)
C7	0.0391 (15)	0.0298 (15)	0.0374 (17)	0.0078 (12)	0.0148 (13)	0.0118 (13)
C8	0.0284 (13)	0.0279 (14)	0.0482 (19)	−0.0110 (11)	0.0146 (13)	−0.0081 (14)
C9	0.0350 (14)	0.0278 (14)	0.0280 (15)	0.0019 (11)	0.0143 (12)	0.0071 (12)

### Geometric parameters (Å, °)

**Table:**

P1—O3	1.5020 (19)	C1—C2	1.534 (3)
P1—O1	1.515 (2)	C2—C3	1.512 (4)
P1—O4	1.552 (2)	C2—H22A	0.9700
P1—O2	1.5713 (19)	C2—H22B	0.9700
P2—O8	1.480 (2)	C3—C4	1.524 (4)
P2—O5	1.5137 (19)	C3—H3	0.9800
P2—O7	1.572 (2)	C4—C5	1.524 (3)
P2—O6	1.582 (2)	C4—H4A	0.9700
O2—H2	0.8200	C4—H4B	0.9700
O4—H4	0.8200	C5—C9	1.526 (4)
O6—H6	0.8200	C5—C8	1.527 (4)
O7—H7	0.8200	C6—H6A	0.9600
O9—H91	0.848 (10)	C6—H6B	0.9600
O9—H92	0.852 (10)	C6—H6C	0.9600
N1—C1	1.523 (3)	C7—H7A	0.9600
N1—C5	1.527 (3)	C7—H7B	0.9600
N1—H1A	0.9000	C7—H7C	0.9600
N1—H1B	0.9000	C8—H8A	0.9600
N2—C3	1.499 (3)	C8—H8B	0.9600
N2—H2A	0.8900	C8—H8C	0.9600
N2—H2B	0.8900	C9—H9A	0.9600
N2—H2C	0.8900	C9—H9B	0.9600
C1—C7	1.525 (4)	C9—H9C	0.9600
C1—C6	1.526 (4)		
			
O3—P1—O1	114.23 (11)	N2—C3—C4	110.5 (2)
O3—P1—O4	107.88 (11)	C2—C3—C4	109.8 (2)
O1—P1—O4	110.16 (13)	N2—C3—H3	108.6
O3—P1—O2	111.20 (12)	C2—C3—H3	108.6
O1—P1—O2	106.82 (12)	C4—C3—H3	108.6
O4—P1—O2	106.28 (12)	C3—C4—C5	111.4 (2)
O8—P2—O5	113.48 (12)	C3—C4—H4A	109.4
O8—P2—O7	108.97 (11)	C5—C4—H4A	109.4
O5—P2—O7	109.72 (12)	C3—C4—H4B	109.4
O8—P2—O6	109.09 (13)	C5—C4—H4B	109.4
O5—P2—O6	109.58 (12)	H4A—C4—H4B	108.0
O7—P2—O6	105.71 (12)	C4—C5—C9	110.8 (2)
P1—O2—H2	109.5	C4—C5—N1	109.09 (19)
P1—O4—H4	109.5	C9—C5—N1	105.1 (2)
P2—O6—H6	109.5	C4—C5—C8	111.7 (2)
P2—O7—H7	109.5	C9—C5—C8	109.7 (2)
H91—O9—H92	114 (3)	N1—C5—C8	110.3 (2)
C1—N1—C5	120.63 (19)	C1—C6—H6A	109.5
C1—N1—H1A	107.2	C1—C6—H6B	109.5
C5—N1—H1A	107.2	H6A—C6—H6B	109.5
C1—N1—H1B	107.2	C1—C6—H6C	109.5
C5—N1—H1B	107.2	H6A—C6—H6C	109.5
H1A—N1—H1B	106.8	H6B—C6—H6C	109.5
C3—N2—H2A	109.5	C1—C7—H7A	109.5
C3—N2—H2B	109.5	C1—C7—H7B	109.5
H2A—N2—H2B	109.5	H7A—C7—H7B	109.5
C3—N2—H2C	109.5	C1—C7—H7C	109.5
H2A—N2—H2C	109.5	H7A—C7—H7C	109.5
H2B—N2—H2C	109.5	H7B—C7—H7C	109.5
N1—C1—C7	105.7 (2)	C5—C8—H8A	109.5
N1—C1—C6	110.8 (2)	C5—C8—H8B	109.5
C7—C1—C6	109.2 (2)	H8A—C8—H8B	109.5
N1—C1—C2	108.6 (2)	C5—C8—H8C	109.5
C7—C1—C2	110.9 (2)	H8A—C8—H8C	109.5
C6—C1—C2	111.5 (2)	H8B—C8—H8C	109.5
C3—C2—C1	111.4 (2)	C5—C9—H9A	109.5
C3—C2—H22A	109.3	C5—C9—H9B	109.5
C1—C2—H22A	109.3	H9A—C9—H9B	109.5
C3—C2—H22B	109.3	C5—C9—H9C	109.5
C1—C2—H22B	109.3	H9A—C9—H9C	109.5
H22A—C2—H22B	108.0	H9B—C9—H9C	109.5
N2—C3—C2	110.7 (2)		

### Hydrogen-bond geometry (Å, °)

**Table:**

*D*—H···*A*	*D*—H	H···*A*	*D*···*A*	*D*—H···*A*
O2—H2···O1^i^	0.82	1.74	2.537 (3)	163
O4—H4···O5^ii^	0.82	1.79	2.538 (3)	152
O6—H6···O3	0.82	1.83	2.646 (3)	172
O7—H7···O1	0.82	1.85	2.662 (3)	173
O9—H91···O5	0.85 (1)	1.97 (1)	2.811 (3)	170 (3)
O9—H92···O3^iii^	0.85 (1)	2.00 (1)	2.837 (3)	165 (5)
O9—H92···O4^iii^	0.85 (1)	2.66 (5)	3.256 (3)	128 (5)
N1—H1A···O8^iv^	0.90	1.84	2.742 (3)	176
N1—H1B···O5^v^	0.90	2.31	3.168 (3)	159
N1—H1B···O8^v^	0.90	2.33	3.038 (3)	136
N2—H2A···O2	0.89	2.05	2.929 (3)	172
N2—H2A···O1	0.89	2.51	3.076 (3)	122
N2—H2B···O3^ii^	0.89	2.07	2.919 (3)	160
N2—H2C···O9	0.89	1.88	2.721 (4)	156
C3—H3···O6^ii^	0.98	2.59	3.406 (3)	141
C4—H4B···O9^vi^	0.97	2.58	3.492 (4)	158
C9—H9A···O7^vi^	0.96	2.40	3.343 (3)	168

Symmetry codes: (i) −*x*+1, *y*+1/2, −*z*+1/2; (ii) −*x*+1, *y*−1/2, −*z*+1/2; (iii) *x*, −*y*+1/2, *z*+1/2; (iv) *x*−1, *y*, *z*; (v) −*x*+1, −*y*+1, −*z*+1; (vi) −*x*+1, −*y*, −*z*+1.
